# Comparative Analysis of Progesterone Secretion and Plasma Proteome Across the Pregnant and Non-Pregnant Luteal Phase of the Koala (*Phascolarctos cinereus*)

**DOI:** 10.3390/proteomes14030040

**Published:** 2026-08-06

**Authors:** Brooke E. Hartley, Stephen D. Johnston, Yolande Campbell, Kerry Fanson, Vere Nicolson, Ashleigh Neal, Taylor Pini

**Affiliations:** 1 School of Veterinary Science, The University of Queensland, Gatton, QLD 4343, Australia; brooke.hartley@uq.edu.au (B.E.H.); t.pini@uq.edu.au (T.P.); 2 School of Environment, The University of Queensland, Brisbane, QLD 4067, Australia; yolande.campbell@uq.edu.au; 3 School of Agriculture, Biomedicine and Environment, La Trobe University, Melbourne, VIC 3086, Australia; k.fanson@latrobe.edu.au; 4 Paradise Country, Gold Coast, QLD 4210, Australia; vere.nicolson@wires.org.au (V.N.); ashleigh_neal@vrtp.com.au (A.N.)

**Keywords:** progesterone, plasma proteome, maternal recognition of pregnancy, marsupial

## Abstract

Background: Koalas are a vulnerable marsupial species with unique reproductive traits. Efforts to develop assisted breeding technologies have been hindered by a limited understanding of maternal recognition of pregnancy and physiological changes induced by the foeto-placental unit. Differences in the reproductive physiology of pregnant and non-pregnant koalas were examined to investigate the possibility of maternal recognition and identify potential pregnancy and/or embryonic loss biomarkers. Methods: Koalas were separated into three groups: pregnant (*n* = 4 cycles from three females), mated but non-parturient (*n* = 4 cycles from three females), and gonadotropin-releasing hormone (GnRH) agonist-treated females (*n* = 7). Plasma was collected on day of mating/GnRH injection (D0) and on multiple subsequent days. Progesterone concentrations were measured by enzyme immunoassay, and plasma proteomes were analysed using filter-aided sample preparation followed by liquid chromatography-tandem mass spectrometry (LC-MS/MS), employing sequential window acquisition of all theoretical fragment ion spectra. Results: Ovulation induction mechanisms influenced peri-ovulatory progesterone secretion (pregnant 40.7 ± 3.3 ng/mL vs. GnRH-treated 15.7 ± 1.8 ng/mL), with no significant differences in progesterone that occurred later in the luteal phase. LC-MS/MS identified 158 proteins, representing the first koala plasma proteome. Leucine-rich alpha-2-glycoprotein (LRG1) was significantly elevated at D2 in pregnant females compared to GnRH-treated females, and pregnant D9 and D19. In pregnant females, fibronectin (FN1) was significantly more abundant at D19 compared to D9 but not significantly different between treatments. Conclusions: These preliminary findings provide foundational data for further investigation into maternal recognition and pregnancy/embryonic loss in koalas.

## 1. Introduction

Koalas (*Phascolarctos cinereus*) are one of Australia’s most iconic and beloved animals with deep-rooted historical, cultural, and economic value. Unfortunately, koala populations are also threatened nationally [1]. While captive breeding programmes can conserve valuable genetic diversity [2], a limitation to genetic management is the koalas’ unique reproductive physiology and the fact that only about half of all pairings result in successful pregnancies [3,4]. As a marsupial, their gestation period is relatively short (~35 days), resulting in a altricial neonate that undergoes further development in the pouch [5,6]. Koalas are seasonally polyoestrous with a defined breeding season (spring to early autumn) [7]. Koalas are also induced ovulators, appearing to require urogenital stimulation and seminal fluid exposure to produce sufficient luteinising hormone (LH) to stimulate ovulation [8]. Therefore, a female may experience three possible reproductive ‘states’ within the breeding season: pregnancy (~35 days from mating to birth), a mated and ovulated but non-pregnant luteal phase (~50 days from mating to the first day of the next oestrus), or a non-ovulatory cycle (~31 days from first day oestrus to the first day of the next oestrus) [5,9].

A key gap in our understanding of koala reproduction is the mechanism underlying maternal recognition. Although early studies of maternal recognition centred on the idea that the presence or absence of an embryo had a direct effect on the lifespan and function of the corpus luteum, the concept has now been further expanded to a variety of other physiological mechanisms by which the embryo might influence the dam [10]. Given that pregnancy does not appear to prolong the lifespan of corpora lutea in marsupials, and that pregnancy is similar in duration to the luteal, it was initially thought that marsupials lacked any form of maternal recognition. However, Renfree [11] has shown clear evidence of maternal recognition in macropodid marsupials. Notably, the uterus secretes certain proteins only when a foetus is present [12]. This discovery has renewed questions as to whether other marsupials, including koalas, may also exhibit maternal recognition.

Interestingly, there is some evidence in koalas that the foetus may impact the function of the corpus luteum, even if it does not extend the lifespan; specifically, there is some evidence that successful matings are associated with higher progesterone concentrations compared with unsuccessful ones [5]. Given progesterone’s role as a potent transcription factor, that difference could be associated with differences in protein abundance in pregnancy. However, a subsequent study that measured faecal progesterone metabolites was unable to differentiate between pregnant and non-pregnant females [9]. Investigating both circulating progesterone levels and protein abundance could provide valuable insights about maternal recognition in koalas.

In addition, there is currently no simple means of early accurate pregnancy diagnosis in koalas that could be used to investigate reproductive failure. Moreover, an ability to detect early pregnancy in the koala would allow a better understanding of embryonic loss. Early, accurate identification of koala pregnancy could potentially be achieved using biomarkers specific to pregnant females. Such biomarkers may reflect physiological changes in pregnancy or be related to maternal recognition of pregnancy (MRP). These biomarkers function as an embryonic signal which alerts the maternal environment to an establishing pregnancy, often with direct luteotropic and anti-luteolytic activities [10], and impacts endometrial function which support embryonic development [13]. While Renfree [12] has previously explored protein changes in uterine fluids of the tammar wallaby during pregnancy, this study was published 50 years ago and identified very few proteins. As a rapidly advancing tool, proteomics may offer insights into koala pregnancy in terms of profiling MRP, systemic maternal changes in response to pregnancy and potential biomarkers for pregnancy diagnosis; for example, previous studies have leveraged proteomics to investigate MRP in horses [14].

Beyond natural mating, protocols for artificial insemination (AI) of koalas have been robustly developed over the last two decades [15,16]. While this technology could provide welfare benefits by limiting animal transportation for breeding, success rates are still highly variable, ranging from 0 to 77% [15]. Currently, AI is timed based on behavioural oestrus; however, controlling ovulation to allow for precise insemination timing may improve success rates, particularly if using cryopreserved semen, which has a shorter lifespan and survival once thawed [15]. Female koalas can be induced to ovulate via a gonadotrophin-releasing hormone (GnRH) agonist, leading to a consequent surge of LH and elevated progesterone in the weeks following injection [17]. However, it is unclear whether this increase in progesterone is identical to that of a mating-induced pregnancy or luteal phase.

Understanding maternal recognition and the impact of the foeto-placental unit on maternal physiology is critical for improving assisted-breeding outcomes in koalas. In addition, it will also help with the development of reproductive biomarkers to monitor pregnancy health. To address this, we investigated whether the foeto-placental unit influences either maternal progesterone levels (as an indicator of luteal function) or protein abundance. We quantified circulating progesterone and protein levels across several key time points in three groups of females (pregnant, mated but non-parturient, and GnRH-treated). This information could potentially contribute to the development of assisted breeding technologies and in the assessment of embryonic loss in koalas.

## 2. Materials and Methods

### 2.1. Animals

This study was conducted using 15 sexually mature healthy captive koalas housed at Paradise Country (Oxenford, Australia) during the 2023 and 2024 breeding seasons (September to April). Koalas were managed under standard husbandry and reproductive procedures as described in Jackson et al. [18]. All procedures were approved by the University of Queensland Animal Ethics Committee on 1 June 2022 (2021/AE001155). Female koalas that were mated in this study were chosen by Paradise Country curatorial staff on the basis of their breeding programme. The females that were not scheduled to be mated during these breeding seasons were selected as the control units (oestrus females that received GnRH only but not mated).

### 2.2. Experimental Design and Sample Collection

There were three treatment groups in this study: (1) koalas that were naturally mated and successfully produced pouch young (pregnant), (2) koalas that were naturally mated but failed to produce pouch young, presumably associated with lack of fertilisation or embryonic loss (mated but non-parturient), and (3) koalas that were induced to ovulate with a GnRH agonist and which received no male contact, so that there was no possibility of a foeto-placental influence (GnRH-treated). Before attempting mating, oestrus was detected using a teaser male and the display of oestrous behaviours as described by Johnston and Holt [2]. Six females were naturally mated over the course of the study with one of five males; two females were mated to different males in both the 2023 and 2024 breeding seasons. Mated females were divided into two groups; those that produced a pouch young ~35 days later (pregnant) (*n* = 4 cycles from 3 females) and those that failed to produce pouch young and returned to oestrus ~50 days later (mated but non-parturient) (*n* = 4 cycles from 3 females). GnRH-treated females (*n* = 7) were used as a control group (no embryo) and to investigate any differences in physiology with those that mated naturally. GnRH agonist (Receptal^®^, 0.0042 mg buserelin acetate equivalent to 0.004 mg of buserelin, MSD Animal Health Biopharma Products, Wellington, New Zealand) was administered via subcutaneous injection to induce ovulation without mating or the presence of semen, as described by Allen et al. [17]. Blood samples (2 mL) were collected from conscious koalas habituated for venipuncture from the cephalic vein using a 23 g × 0.75” winged infusion set (Terumo Australia Pty Ltd., Sydney, NSW, Australia) and 3 mL syringe (Terumo Australia Pty Ltd., NSW, Australia) [19]. Blood samples were collected on the day of mating or GnRH agonist injection (D0). Additional samples were subsequently drawn on multiple days post mating or injection for the length of gestation (~D35) or the luteal phase (~D50). Blood was centrifuged (10,000× *g* for 5 min) to isolate plasma, divided into two aliquots and stored at −80 °C. Progesterone concentration was assessed in all collected samples. Proteomic analysis was performed on the same *n* = 3 individuals per treatment group, per time point. Females chosen for proteomics were selected based on if their sampling was complete or mostly complete (e.g., up to D55 for GnRH-treated) and as consistent as possible (e.g., every 5 days).

### 2.3. Progesterone Enzyme-Immunoassay (EIA)

Progesterone EIA kits (Arbor Assays^TM^, #ISWE003, Ann Arbor, MI, USA) were used to determine plasma progesterone concentration as per the manufacturer’s protocol. Progesterone concentrations were determined using duplicate technical replicates. Progesterone was then read using a microplate reader (BMG Labtech, Victoria, Australia; SpectroStar Nano Plate Reader, 450 nm measuring filter with 620 nm reference filter) with MARS data analysis (BMG Labtech, Victoria, Australia). High- and low-quality controls ran at ~30% and 80% binding. Intra-assay coefficients of variation (CV) for both controls were <10%. Inter-assay CVs for controls were 13.6% (high) and 8.9% (low). All samples were run in duplicate, and all of an individual’s samples were run in the same batch. Concentration is expressed as ng/mL of plasma.

### 2.4. Progesterone Data Analysis

All data analysis was performed in R Studio (Posit^®^, v2025.05.1; Boston, MA, USA). Blood sampling days were sorted into the following time bins: D0, D1, D2—3, D4—7, D9—12, D14—17, D19—21, D23—26, D28—32, D34—36, D39—42, and D45—46; they are subsequently referred to as time points D0, 1, 2, 5, 10, 15, 20, 25, 30, 35, 40, and 45, respectively.

To determine if there was a significant difference in total progesterone output between treatment groups, the area under the curve (AUC) was calculated for each female (pracma package v2.4.5). The curve was standardised to start at D0 and end at D35 to account for less sample days in the pregnant treatment. A one-way ANOVA was used to test for differences between groups, with AUC as the response variable and treatment group as the predictor variable.

To more closely examine differences between the treatment groups at specific time points, progesterone concentrations were analysed using a linear mixed-effects model with restricted maximum likelihood (*lme4* package v1.1-37). Treatment, time point (entered as a categorical factor), and their interaction were specified as fixed effects. Female ID was included as a random effect to account for repeated sampling. Model assumptions were evaluated by inspecting residual diagnostics. Post hoc pairwise comparisons were conducted using Tukey Honest Significant Difference (*lmerTest* v3.1-3 and *emmeans* v2.0.3 packages). Differences were considered significant if the adjusted *p*-value was <0.05.

### 2.5. Filter-Aided Sample Preparation (FASP) for Proteomic Analysis

Plasma samples from D0, D2, D9, D19, and D34 from pregnant (*n* = 3), mated but non-parturient (*n* = 3) and GnRH-treated (*n* = 3) females were employed for proteomic analysis. No technical replicates were included for proteomic analysis. Plasma samples were treated with Sigma complete protease inhibitor (1:10 *v*/*v*) and protein concentration determined by bicinchoninic acid assay (BCA) (Pierce™ BCA Protein Assay Kit 23,225, Thermo Fisher Scientific, Rockford, IL, USA). Samples were prepared for liquid chromatography-tandem mass spectrometry (LC-MS/MS) using filter-aided sample preparation (FASP) [20]. The 30 kDa molecular weight cut-off spin filters (MWCO; UFC5030, Amicon Ultra, Merck, Melbourne, Australia) were washed with 8 M of urea in 50 mM ammonium bicarbonate (UA) (13,000× *g*, 15 min), and 20 μg of protein per sample was added to spin filters (13,000× *g*, 15 min), prior to washing once with UA (13,000× *g*, 15 min). Samples were reduced (10 mM dithiothreitol, room temperature, 30 min) and alkylated (50 mM, 30 min, room temperature in the dark). Filter units were then centrifuged (13,000× *g*, 10 min) before being washed with 8 M urea (3×, 13,000× *g*, 15 min) and 50 mM ammonium bicarbonate (3×, 13,000× *g*, 10 min). After washing, filter units were transferred into new collection tubes and treated with trypsin with 0.01% (*v*/*v*) ProteaseMax (Promega, V5113, V2071, Madison, WI, USA) at 1:50 (trypsin:total protein dilution) and incubated overnight at 37 °C. Filter units were centrifuged (13,000× *g*, 10 min) and peptides eluted with 0.5 M sodium chloride (13,000× *g*, 10 min). C18 ziptips (Millipore, ZTC18S096, ~5 μg binding capacity, Merck Life Science Pty Ltd, Melbourne, Australia) were equilibrated with 80% acetonitrile and 0.1% formic acid (*v*/*v*), then 1% acetonitrile and 0.1% formic acid (*v*/*v*). Then, 10 μL of sample was loaded into ziptips, before washing with 1% acetonitrile and 0.1% formic acid (*v*/*v*). Washed peptides were eluted with 80% acetonitrile and 0.1% formic acid (*v*/*v*). Prior to injection, peptides were dried by vacuum centrifuge to remove acetonitrile and resuspended in 0.1% formic acid (*v*/*v*).

### 2.6. LC-MS/MS Analysis with Sequential Window Acquisition of All Theoretical Fragment Ion Spectra (SWATH) Acquisition

Peptides were separated by reversed-phase chromatography on a Waters M-Class UPLC system with Waters NanoEase HSS T3 column (100 A, 1.8 µm, 300 µm × 150 mm). Chromatography was operated at 5 μL/min and column set at 40 °C with liquid chromatography (LC) conditions, 0–0.6 min = 5% B, 0.6–22 min = 5–35%, 22–23 min = 35–90%, and held at 90% for 3 min followed by re-equilibration for 4 min. Pump A = 0.1% formic acid (*v*/*v*) in water, and pump B = 0.1% formic acid (*v*/*v*) in acetonitrile. Eluted peptides were directly analysed on ZenoTof 7600 instrument (ABSciex) using Optiflow Micro/Micro Cal source. Curtain gas = 35 psi, CAD gas = 7, Gas 1 = 20 psi, Gas 2 = 15 psi, source temp = 150 °C, spray voltage = 5000 V, DP = 80, and CE = 10. Data-dependent acquisition using mass spectrometry (MS) time-of flight (TOF) 400–1500 m/z scanning was performed for 0.2 s, followed by data-dependent acquisition of up to 20 peptides with intensity > 150 counts, across 100–2000 m/z windows (0.035 s) using dynamic collision energy. Zeno pulsing was on and threshold set to 100,000 cps. Zeno-SWATH acquisition occurred using MS TOF scan across 400–1500 m/z (0.1 s). For MS2, variable windows spanning 399.5 m/z–750.5 m/z were selected for fragmentation (0.013 s) with fragmented data acquired across 140 to 1750 m/z. Zeno pulsing was on and threshold set to 100,000 cps and dynamic collision energy was used.

### 2.7. Protein Identification, Quantitative Comparison and Bioinformatic Analysis

Identification and quantification of proteins was conducted using DIA-NN [21] (v1.9.2). Raw (Wiff) files were converted to MzML using Proteowizard. An in silico ion spectral library was generated using the reference koala proteome from Uniprot (FASTA file downloaded May 2025). Proteins were identified against the in silico library using standard DIA-NN analysis settings; a minimum of two peptides was required for identification. Scoring was performed in the ‘generic’ mode and protein inference used the default ‘genes’ setting, prioritising protein level quantification over confidence identification of proteoforms and paralogues. DIA-NN output was converted to an *MSstats* (v 2.20.0) input file using *MSstats_labelfree_preprocessing* (https://github.com/MiguelCos/MSstats_labelfree_preprocessing, accessed 22 May 2025) in R Studio. From this, the greatest abundant proteins could be identified by calculating the average median intensity of proteins across samples (MS Excel). Principal component analysis (PCA) was used to explore global proteome visual similarity across individual samples. Global PCA plots were generated in R studio (v 2025.05.1) using *FactroMineR* (v 2.12), *factoextra* (v 1.0.7), *missMDA* (v 1.20), and *ggplot2* (v 3.5.2). PCA plots for D2 and D34 were also generated, as these were days that showed differences in progesterone concentration and/or where differences associated with gestation/parturition were most likely to occur.

To perform filtering and statistical analysis across treatments, the *MSstats* (v 2.20.0) [22] package was employed. The dataProcess function was used to normalise the data, with analysis across groups performed using groupComparison. Groupwise comparisons were performed both across treatments at each time point (e.g., pregnant D0 v GnRH-treated D0) and across time points within each treatment (e.g., mated but non-parturient D0 v D19). Proteins were considered significantly different if the adjusted *p*-value was <0.05 and log2 fold change > |0.6|. A more relaxed criterion was employed to explore potential differences across the three treatments at D2 and D34. This test employed the unadjusted *p*-value, which does not take into consideration statistical correction, and was used as a hypothesis-generating tool. Proteins were considered different if the unadjusted *p*-value was <0.05. Volcano plots comparing the differences found using the unadjusted *p*-value were generated using *tidyverse* (v 2.0.0), *ggplot2* (v 3.5.2), *ggrepel* (v 0.9.6) and *RColorBrewer* (v 1.1-3) packages. Proteins which were absent in all biological replicates of one treatment group and present in at least two of three biological replicates in the comparator group were described as “detected only in one treatment”.

Both significantly different proteins and the 15 most abundant proteins were analysed by gene ontology using DAVID (Database for Annotation, Visualization, and Integrated Discovery, v 6.8). Annotation results grouped by molecular function were considered significant if the enrichment score was >3.0. Poorly described or koala-specific proteins were explored using Uniprot BLAST (https://www.uniprot.org/blast, accessed 13 August 2025); homology > 80% was considered to indicate a homologous protein described in another species.

## 3. Results

### 3.1. Differences in Progesterone Following Mating Versus GnRH-Induced Ovulation

Total progesterone output during pregnancy/luteal phase did not differ significantly between treatments (*p* = 0.673). AUC for each biological replicate varied from 21.14 to 38.52 ng/mL. While there was no significant difference between treatments, mean progesterone AUC was slightly lower for pregnant females (25.78 ± 3.55 ng/mL) than for both mated but non-parturient (29.31 ± 8.09 ng/mL) and GnRH-treated AUC (28.65 ± 5.74 ng/mL).

**Figure 1:**
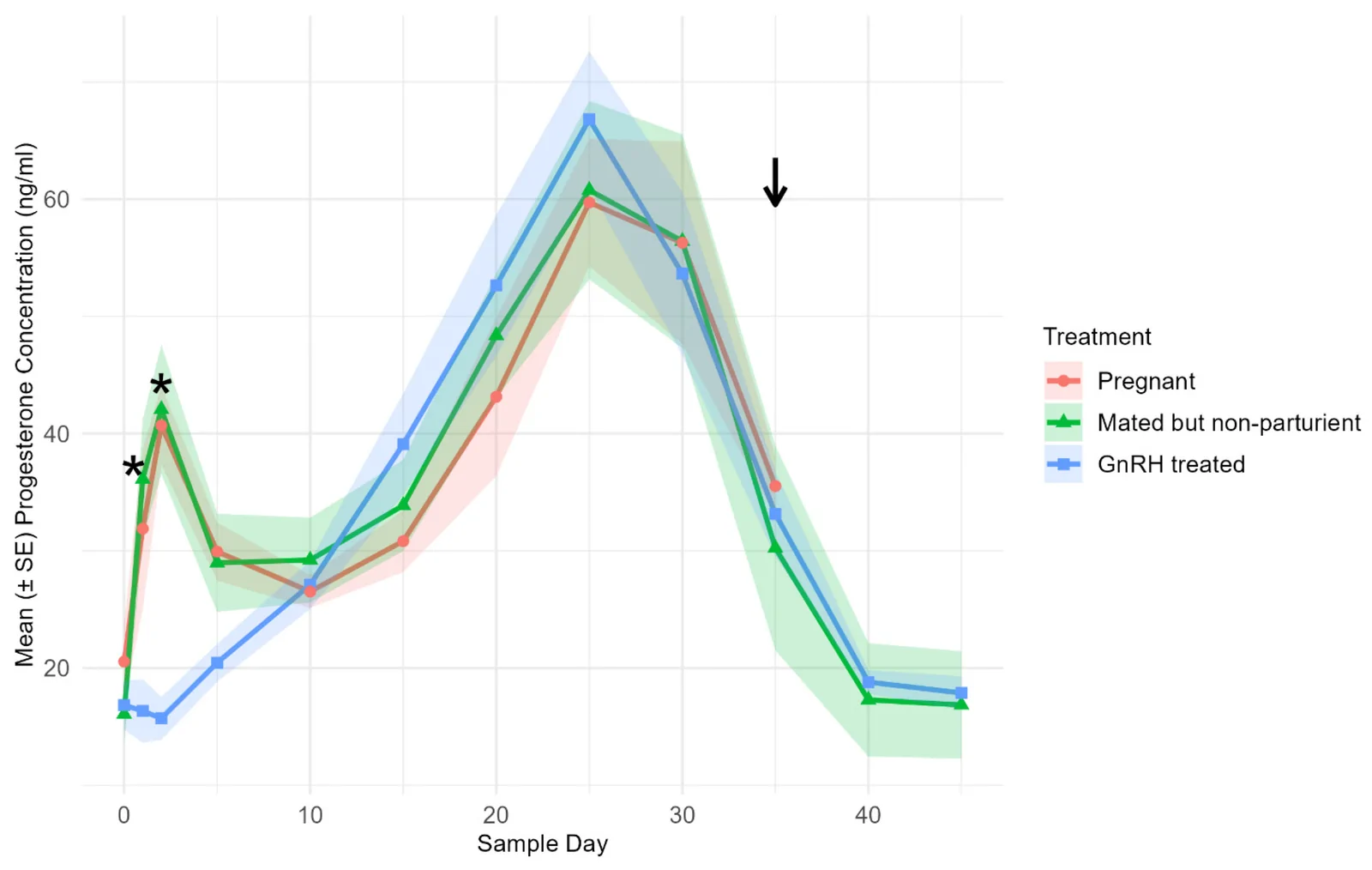
Mean (± SE; indicated by shading) progesterone concentration (ng/mL) for the gestational period in pregnant koalas (*n* = 4 cycles from 3 females; ~35 days; ↓ indicating birth) and for the luteal phase (45 days) in mated but non-parturient (*n* = 4 cycles from 3 females), and GnRH-treated koalas (*n* = 7); * indicates significant difference between treatments (*p* < 0.05).

At a finer scale, the results of the linear model revealed a significant interaction between treatment and time point (*p* = 0.007). Specifically, progesterone concentrations in naturally mated females (both pregnant and non-pregnant) increased from D0 to D2, before decreasing slightly at D5 (Figure 1). However, this pattern was not reflected in GnRH-treated females as progesterone remained low at D2 before increasing at D5. Post hoc comparisons indicated that GnRH-treated females had significantly lower progesterone levels than the other two treatment groups between days 1–3 (all *p* < 0.05). After D5, all koalas showed a similar increase in progesterone until a peak at D25 (Table 1). Progesterone levels then decreased to basal levels at D40, prior to return to oestrus or following parturition.

### 3.2. Differences in the Plasma Proteome Reflect Both Successful Pregnancy and Ovulation Mechanism

**Figure 2:**
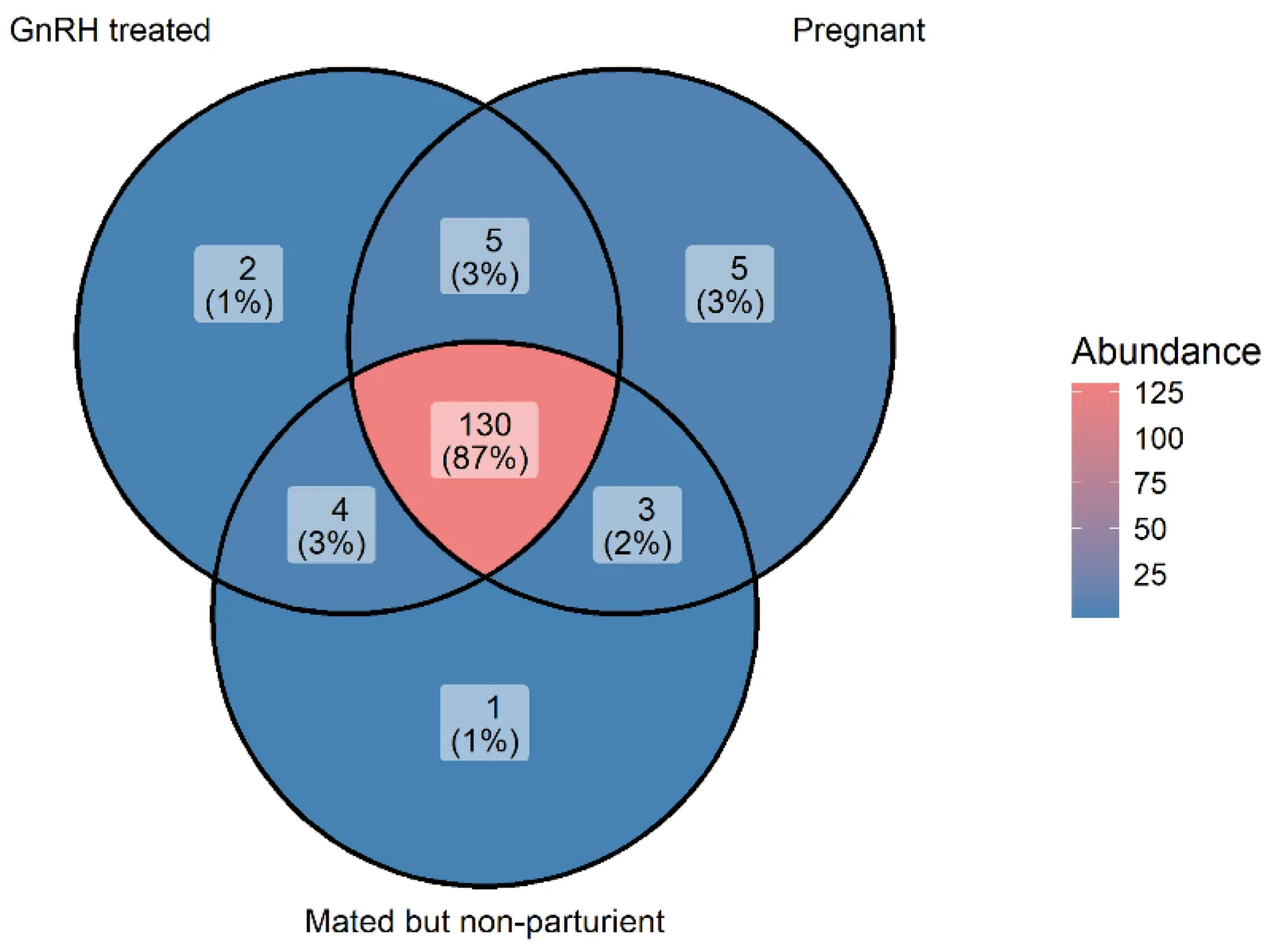
Venn diagram of proteins found in two or more biological replicates discovered in koala plasma of pregnant (*n* = 3), mated but non-parturient (*n* = 3), and GnRH-treated (*n* = 3) females.

A total of 158 proteins were identified across the three treatment groups (Supplementary Table S1), with 150 proteins found in two or more biological replicates (Figure 2). The top 15 most abundant proteins (Table 2) were highly enriched in serum albumins (DAVID enrichment score 4.55) and fibrinogens (DAVID enrichment score 3.83).

**Figure 3:**
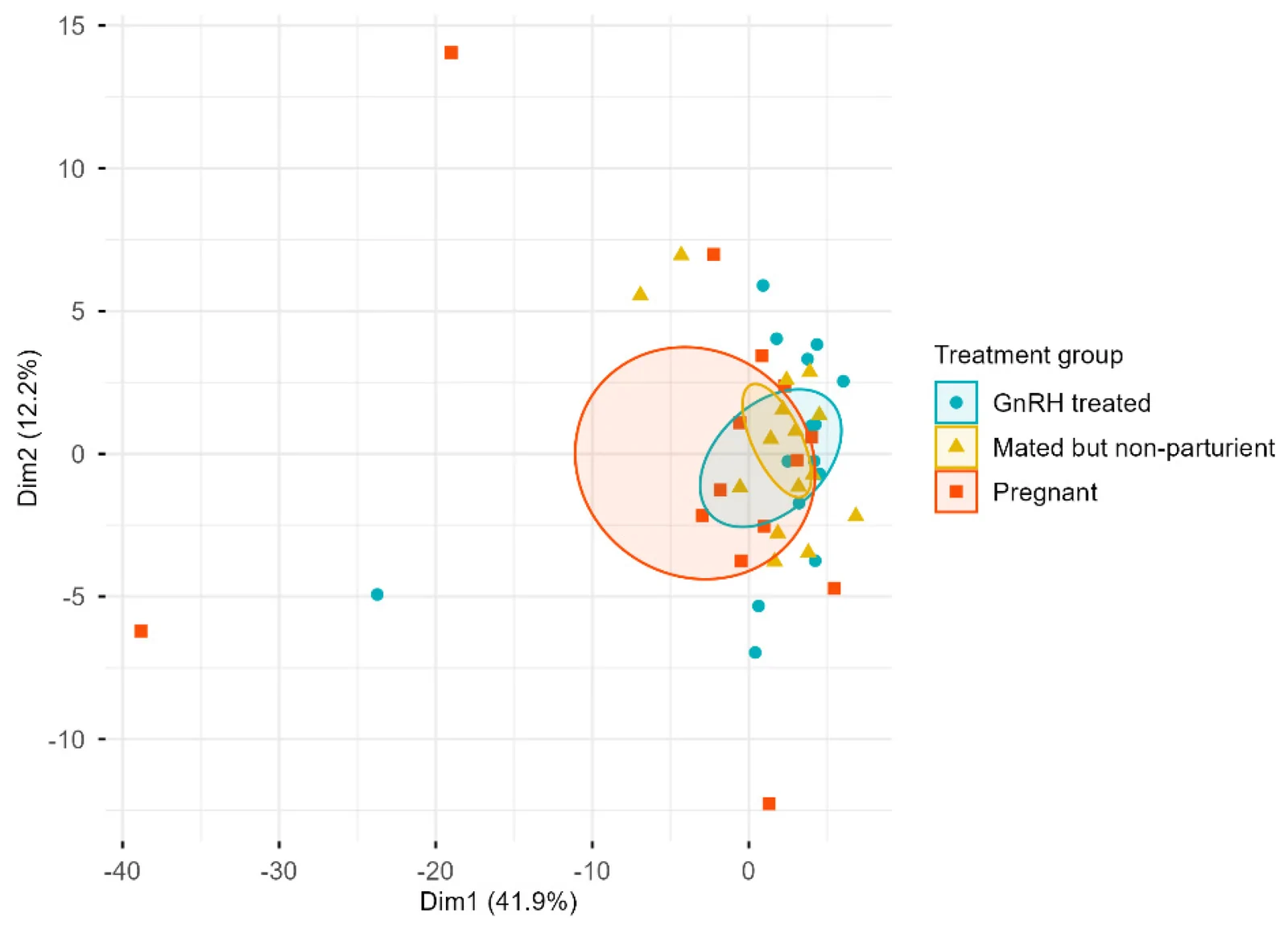
Principal component analysis of proteomic data from GnRH-treated (*n* = 3 per time point), mated but non-parturient (*n* = 3 per time point), and pregnant (*n* = 3 per time point) koala plasma at D0, 2, 9, 19 and 34 post mating/GnRH injection.

PCA of the global proteome across all time points revealed no apparent separation based on treatment; however, four outliers were identified (Figure 3). Three of the four outliers originate from two pregnant females on D2, 9, and 34, whereas the remaining outlier was a GnRH-treated female on D34. A PCA at D2 post mating/injection showed visual separation of pregnant compared to mated but non-parturient and GnRH-treated plasma proteomes, whereas at D34, which is typically 1 day before the expected time of parturition, it revealed that there was no obvious separation of the three treatments (Supplementary Figures S1 and S2).

Two proteins were identified as candidate proteins that require further investigation (adjusted *p*-value < 0.05, fold change > |0.6|). Using an adjusted *p*-value these proteins differed either between or within treatment comparisons. At D2, leucine-rich alpha-2-glycoprotein (LRG1) was significantly more abundant (adjusted *p* = 0.01) in the pregnant females (12.66 ± 0.12) compared to the GnRH-treated females (11.11 ± 0.41), but the not mated but non-parturient (11.68 ± 0.15) koalas (Supplementary Table S2). Additionally, LRG1 was significantly more abundant (adjusted *p* = 0.01) in the pregnant treatment at D2 (12.66 ± 0.12) compared to the same pregnant koalas at D9 (11.14 ± 0.004) and D19 (11.07 ± 0.5).

Further, associated with elevated progesterone secretion at D19, fibronectin (FN1) was significantly elevated (adjusted *p* = 0.047) in the pregnant treatment (15.06 ± 1.94) compared to D9 of pregnancy (11.23 ± 1.34). While FN1 was also higher in pregnant koalas compared to non-parturient (12.48 ± 0.18) and GnRH-treated koalas (13.11 ± 0.18) at D19, this difference was not statistically significant (Supplementary Table S3).

**Figure 4:**
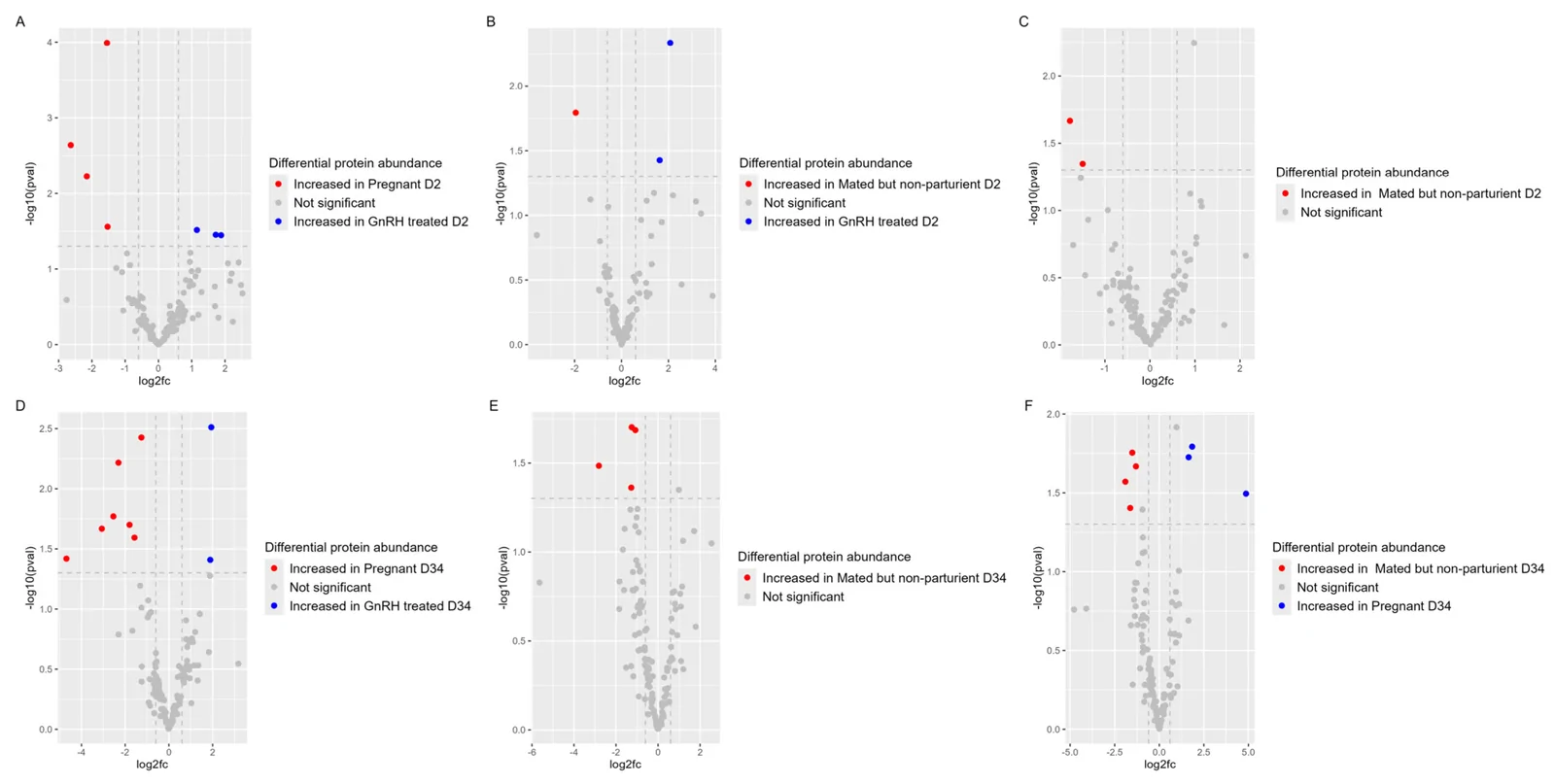
Volcano plots (−log10 *p*-value vs. log2 fold change) of pairwise comparisons for (**A**) pregnant D2 (*n* = 3) vs. GnRH-treated D2 (*n* = 3), (**B**) mated but non-parturient D2 (*n* = 3) vs. GnRH-treated D2 (*n* = 3), (**C**) mated but non-parturient D2 (*n* = 3) vs. pregnant D2 (*n* = 3), (**D**) pregnant D34 (*n* = 3) vs. GnRH-treated D34 (*n* = 3), (**E**) mated but non-parturient D34 (*n* = 3) vs. GnRH-treated D34 (*n* = 3), and (**F**) mated but non-parturient D34 (*n* = 3) vs. pregnant D34 (*n* = 3) proteins. Dotted lines indicate applied significance thresholds using a less restrictive statistical test (unadjusted *p*-value). Coloured dots indicate an increase in abundance of proteins in different treatments using an unadjusted *p*-value and grey dots represent proteins with no significant difference between treatments.

As a lack of significantly different proteins were identified using a strict statistical test, a more relaxed criterion was applied to explore proteins that may prove to be significant with further research. Possible differences in protein abundance between the three treatments at D2 and D34 were investigated using this relaxed statistical test. Volcano plots that employed an unadjusted *p*-value for D2 showed that there appeared to be an increase in abundance of four proteins in the pregnant treatment in comparison to the GnRH-treated females (Figure 4A). However, only one protein showed a possible increase in abundance in mated but non-parturient treatment at D2 than GnRH-treated D2 (Figure 4B). In a comparison between mated but non-parturient and pregnant females at D2, two proteins demonstrated an apparent increased abundance in the mated but non-parturient treatment while none showed an elevated abundance under exploratory analysis in the pregnant treatment (Figure 4C). In addition, one day prior to the expected parturition time (D34), pregnant females appeared to show a higher abundance in seven proteins in comparison to GnRH-treated females (Figure 4D), while mated but non-parturient females had four proteins with an apparent increase in abundance compared to GnRH-treated females (Figure 4E). Lastly, pregnant females showed three proteins which had a noticeable increase in abundance compared to mated but non-parturient females at D34 under exploratory analysis (Figure 4F).

Proteins which were not detected in any biological replicate of one treatment/time point and detected in at least two biological replicates of the comparative treatment/time point were classified as being present only in one treatment/time point (Supplementary Table S3). tRNA methyltransferase 11 (TRMT11) and serum amyloid A-like protein (LOC110218991) were detected in naturally mated treatments but not the GnRH-treated at D2. Lumican (LUM) was detected in both the mated but non-parturient and the GnRH-treated, but not in the pregnant treatment at D9. Similarly, superoxide dismutase 3 (SOD3), ADAMTS-like 3 (ADAMTSL3), integrin subunit beta-like 1 (ITGBL1), selenoprotein P (SELENOP), and lipopolysaccharide-binding protein (LOC110211440) were detected in the mated but non-parturient treatment but not in pregnant or GnRH-treated at D9.

At the time of the initial progesterone spike in naturally mated koalas (D2), complement C1q C chain (C1QC), glycosylphosphatidylinositol-specific phospholipase D1 (GPLD1), family with sequence similarity 43 member B (FAM43B), carbonic anhydrase 2 (CA2), ficolin 2 (FCN2), serum amyloid P-component-like protein (LOC110201860), and serum amyloid A-like protein (LOC110218991) were detected in pregnant females but were not detected at D0, 9, or 19. Additionally, serum albumin-like protein (LOC110209799) was detected in pregnant females at D2 but not D0 or 19. A comparison of D2 and 9 in the pregnant treatment revealed that proteins detected only on D2 were enriched in secreted/signal/extracellular space components (DAVID enrichment score 4.74).

## 4. Discussion

This study explored progesterone secretion and the accompanying circulating proteins of the luteal phase across three groups of koalas; females that had successful pregnancies, the mated but non-parturient, and koalas that were chemically induced into a luteal phase by administration of 4 μg/kg dose of a GnRH agonist. Despite previous koala studies that showed differences in progesterone secretion between animals that successfully gave birth and those that failed following natural mating [5], the current study showed no significant difference in the overall progesterone profiles between these cohorts, even when compared to koalas that were induced into a luteal phase with the GnRH agonist. This overall lack of difference in progesterone secretion suggests that the koala embryo/foeto-placental unit appears to have little to no impact on corpora luteal function and suggests that differences expressed in the previous study [5], were more likely associated with individual animal variability.

While natural mating resulted in a short-lived progesterone pulse at D2 prior to a gradual increase, this pulse was clearly absent in the GnRH-treated females; this was in fact the only significant difference in progesterone secretion we identified across gestation and luteal phases. A short-lived progesterone spike promptly after mating has been observed in a range of marsupials [35,36], particularly those with longer oestrous cycles. Such a pulse has been associated with transformation and luteinisation of the ovulated follicle to a CL and/or the activation of the CL in those macropod species that exhibit embryonic diapause. For example, the quokka (*Setonix brachyurus*) exhibits a small progesterone pulse that is seen only in pregnant females, potentially regulated by blastocysts [37]. Hinds and Tyndale-Biscoe [38] also detected an initial progesterone pulse in the tammar wallaby (*Macropus eugenii*) in both pregnant and non-pregnant females who had recently ovulated. They asserted that this progesterone ‘pulse’ was released by the newly luteinised CL to stimulate uterine secretions, supporting embryonic development prior to development of a fully functional CL [35,36]. Given the lack of any strong evidence for embryonic diapause in the koala, an earlier pulse of progesterone associated with luteinisation seems more plausible.

A number of studies have recently alluded to an unusually high proportion of interstitial tissue in the koala ovary [38,39] that have shown that this tissue has the capacity for steroidogenesis. As has been described in rabbits (*Oryctolagus cuniculus*) [40,41], it is possible that following the LH surge, which occurs 24–32 h post mating [8], koala ovarian interstitial tissue may also be a source of the progesterone metabolite 20α-hydroxypregn-4-en-3-one (20α-OH), thereby contributing to the initial pulse of the luteal phase. Studies of the rabbit [42] have also highlighted the role of prolactin in maintaining the steroid-producing capacity of this tissue, but no such endocrine studies of prolactin have yet been conducted through gestation in the koala.

The lack of a progesterone pulse in the GnRH-treated females at D2 could possibly mean that the initial spike is related to the physical mechanism of ovulation induction in naturally mated koalas, or perhaps the presence of ovulation-stimulating factors in the semen [8]. In rodents that experience induced ovulation, there is some evidence that the adrenal gland may contribute to the peri-ovulatory progesterone spike, either directly through the release of adrenal progestogens, or indirectly by stimulating the development of the corpus luteum (e.g., bank vole (*Myodes glareolus*) [43]). Cross-talk between adrenal glands and gonads stimulated by mating could also contribute to the underlying mechanism responsible for the progesterone spike seen in koalas.

Regardless, the mechanisms underlying the peri-ovulatory progesterone spike may have implications for captive koala breeding, particularly artificial insemination (AI). While Johnston et al. [44] have successfully used GnRH induction followed by AI to generate viable koala joeys, only one of eight females produced a pouch young. This low success rate may be associated with the absence of a peri-ovulatory progesterone spike in GnRH-treated females. Further investigation into the mechanisms driving this progesterone spike may improve our captive breeding programmes employing AI.

This study appears to be the first to explore the koala plasma proteome, identifying 158 proteins, and represents a similar depth of analysis to recently published plasma proteomes in non-model species [45,46]. Highly abundant plasma proteins included common mammalian plasma proteins (e.g., fibrinogen gamma-, beta, and alpha-chains (FGG, FGB, and FGA), histidine-rich (HRG) and alpha-2-HS (AHSG) glycoproteins). Roughly 50% of the top abundant proteins are involved in the innate immune response, particularly in inflammation, with other proteins of interest involved in pregnancy and venom protection.

To explore koala proteomes further, principal component analyses of samples and volcano plots using an unadjusted *p*-value were conducted. This was used as an exploration tool solely and it did not employ a strict statistical test. While PCA across the complete luteal phase/gestation revealed no visual separation, at D2, it did demonstrate an apparent separation of the pregnant treatment from the non-pregnant treatments. Volcano plots using an unadjusted *p*-value revealed that some proteins demonstrated an apparent increase in abundance at D2 and D34. These exploratory findings indicate that with further targeted research on a larger scale, we may see more differences between the pregnant and non-pregnant treatments, as well as the naturally mated and GnRH-treated females.

In comparisons between treatments employing a strict statistical test using an adjusted *p*-value, only leucine-rich alpha-2 glycoprotein 1 (LRG1) was found to be significantly different at D2. Interestingly, fibronectin (FN1) was elevated between D2 and D9 in pregnant koalas but was not significantly different to FN1 levels at D9 when compared to mated but non-parturient or GnRH-treated koalas.

Using an adjusted *p*-value, leucine-rich alpha-2 glycoprotein 1 (LRG1) was elevated in pregnant koalas at D2 compared to pregnant koalas in D9 and D19, and GnRH-treated koalas at D2. LRG1 is known to aid in cell adhesion and development, regulate angiogenesis, and promote wound healing [47]. Koala mating can cause trauma to the female urogenital sinus, as the glans penis has keratinised spines presumably to increase mechanical reflex stimulation [16]; hence, elevated LRG1 seen in pregnant koalas shortly after mating could potentially be a response to the superficial physical trauma of the urogenital sinus, although one might also expect this protein to also be elevated in mated but non-parturient koalas, but this was not the case. Considering that LRG1 abundance is heightened in the pregnant treatment during early gestation, it may also be related to successful ovulation or fertilisation. If this is the case, reduced abundance of LRG1 in the GnRH-treated females may mean this method of induction of the luteal phase may be comprising ovulation physiology. There may be a connection between a reduced abundance of LRG1 and lack of progesterone spike at D2 in the GnRH-treated females. Therefore, reduced abundance of LRG1 may present a challenge for procedures such as artificial insemination when using GnRH to facilitate ovulation. While our findings cannot confirm LRG1 as a potential biomarker, it does suggest it may be a candidate protein for further validation. A targeted exploration of changes in LRG1 concentration partnered with an investigation of progesterone within the first 72 h post mating/injection in naturally mated versus pharmacologically ovulated koalas is therefore warranted.

Fibronectin (FN1) was detected in the pregnant treatment at D19 and was significantly higher compared to D9 of pregnancy. FN1 has been shown to be highly abundant in uterine tissue of the grey short-tailed opossum (*Monodelphis domesticus*) during late gestation, though its function is unknown [48]. In eutherian mammals, FN1 is involved in oocyte maturation, fertilisation and placentation during early gestation [48,49,50]. While FN1 is still present throughout the luteal phase in mated but non-parturient and GnRH-treated koalas, it tended to be lower in abundance but not statistically significantly compared to pregnant females at D19. Thus, it does not currently present as a potential biomarker of pregnancy in koalas. Further targeted validation of FN1 and investigation into its role in koala embryonic development may be valuable, given its contribution in eutherian mammalian pregnancy.

Highly abundant koala plasma proteins in our analysis also included a protein known to be involved in pregnancy, alpha-2-macroglobulin (A2M). A2M, a hepatic glycoprotein, is involved in placental vascular endothelial growth in humans and rats [51]. While it has a role in regulating trophoblastic invasion during implantation in rodents [52], observation of highly abundant A2M in koalas was not restricted to pregnant females. Like in many other mammals, A2M may also be involved in the innate immune system of koalas as an endoprotease inhibitor [51,53]. As A2M is the most abundant protease inhibitor in healthy human plasma and a biomarker of disease [54,55,56], it may be worth investigating the abundance of A2M in koalas of divergent health status. If there is a significant decreased abundance of A2M in koalas with known disease, A2M may be useful as a biomarker of ill health in captive populations, which would ultimately assist in selectively breeding healthy individuals.

Serum amyloid A (SAA)-like protein (LOC110218991), serum albumin-like protein (LOC110209799), and lumican (LUM) have all been previously identified during pregnancy in other mammalian species. In our study, SAA-like protein was detected only in naturally mated treatments but not GnRH-treated at D2, likely when ovulation occurs. While moderate levels of SAA aids placentation in women by regulating trophoblast invasion and metalloprotease activity [57], only superficial direct contact of the koala conceptus with the uterine epithelium occurs late in gestation [58]. While our observations suggest that SAA-like protein may instead be related to differences between mechanisms of ovulation, similarly to the progesterone spike, further research is necessary.

Furthermore, serum albumin-like protein was detected in pregnant females at D2 but not D0 or D19. Renfree [12] has also detected a serum albumin-like protein in tammar wallaby uterine fluid during early gestation. Although its function has not yet been explored in marsupials, in women, serum albumin supports blastocyst growth [59] and acts as a steroid carrier, inhibited by alpha-fetoprotein as pregnancy progresses [60,61]. Further investigation into potential roles of serum albumin in marsupial pregnancy may provide insight into its temporal changes in abundance.

Lastly, LUM was detected only in non-pregnant treatments at D9. Decreased abundance of LUM may be associated with preeclampsia in women, by inhibiting cell proliferation [62]. However, we saw no indication that the absence of LUM impacted the dam or foetus in koalas.

We also observed venom metalloproteinase inhibitor DM43-like protein (LOC110197834) in high abundance. It has high homology (60.6%) to the Southern opossum (*Didelphis marsupialis*) venom metalloproteinase inhibitor DM43. We hypothesise that LOC110197834 may similarly inhibit the haemorrhagic and apoptotic effects of snake venom metalloproteinases (SVMP) [30,63]. While SVMP inhibitors have been identified in other American opossums [64], this is the first identification in an Australian marsupial, potentially suggesting an evolutionarily conserved protein in marsupials. Mammalian metalloproteinases are involved in inflammation and wound healing; however, increased abundance of metalloproteinases can be harmful [61,65]. By regulating metalloproteinase, inhibitors reduce the risk of deleterious effects [61]. It is possible that the venom metalloproteinase inhibitor-like protein found in this study may not be directly related to inhibition of SVMP, but instead regulate endogenous metalloproteinases.

Many proteins identified in only one treatment or time point are considered common mammalian plasma proteins and include C1QC, FCN2, and serum amyloid P-component-like protein (LOC110201860), all of which have roles in the complement system and inflammation [66,67,68,69]. TRMT11, GPLD1, FAM43B, and CA2 are serum proteins typically regulated by the liver to aid with physiological processes associated with enzyme function, glucose metabolism, cell growth, and carbon dioxide reversible hydration [70,71,72,73].

While our study has presented novel insights into koala pregnancy, as a preliminary study in a non-model species, there are limitations worth noting. Firstly, there is poor annotation of the koala genome. This study relied heavily on in silico spectral librarian information on protein functions and is largely based on protein homology. Inferences into their possible roles in pregnancy is therefore speculative and requires future validation. We are further restricted by whether the mated but non-parturient females experienced fertilisation failure or embryonic loss. While we aimed to provide some insight into embryonic loss in koalas, the experimental design does not fully allow us to confidently claim that protein differences between the mated but non-parturient and the pregnant females—particularly during early pregnancy—occurred due to the presence or absence of an embryo. Currently, there is no minimally invasive and reliable means of distinguishing between fertilisation failure or embryonic loss in the mated but non-parturient females. Similarly, it is difficult to fully separate out the pregnancy-specific effects from those of mating or ovulation when comparing pregnant and GnRH-treated females, especially during the peri-ovulatory period. It is possible that proteins with differing abundance may be a result of mating, seminal exposure, or stimulation in the pregnant group versus GnRH-treated group, rather than fertilisation or the presence of an embryo.

Working with wildlife, especially endangered species where there is limited understanding of reproductive physiology, means that precisely controlling experimental design is challenging. This study utilised captive koalas in a commercial wildlife park, so the sampling schedule needed to be managed to fit in the daily activity of zoo-keeping staff. In addition, we currently have no method for synchronising oestrus in the koala, so that koalas come into oestrus independently. To account for this asynchrony, a time binning system was implemented for our analysis. This may have obscured biologically relevant temporal variations. Furthermore, our sampling schedule for proteomic analysis focused only on major pregnancy milestones, rather than throughout the whole of the luteal phase. Future research may benefit from increased sampling post ovulation and fertilisation to investigate differences in protein abundance during early pregnancy in higher resolution.

As a preliminary study on a novel species, there was a small number of biological replicates with no proteomic technical replicates. While this reduces the statistical power of our findings, we need to be conscious that we are dealing with endangered wildlife, which have their own inherent limitations regarding intensive sampling. Additionally, as a non-model species, there are limited depletion reagents for koalas. Thus, more abundant proteins like albumin and immunoglobins were retained. This likely limited the number of unique proteins detected and therefore candidate proteins identified. Moreover, there was no targeted confirmation of candidate circulating proteins; this could be a key tool which may reveal valuable insights into our candidate proteins for pregnancy and/or embryonic loss.

Lastly, the identification of proteoforms and paralogues was not included in this study. As the first proteome to be investigated in the koala, we prioritised protein level quantification over confidence identification of proteoforms. However, this may have led to misguided functional inferences of identified proteins and important biological information may have been missed. Given the importance of proteoforms in determining functional biomarkers, further investigation into proteoforms of candidate circulating proteins is warranted.

## 5. Conclusions

This study has provided the first investigation into differences in progesterone production between naturally mated and exogenously induced ovulation/luteal phase, as well as changes in circulating proteins during gestation and the non-pregnant luteal phase in koalas. While we found that the mechanism of luteal phase induction influences peri-ovular progesterone secretion during early gestation/luteal phase, progesterone secretion in pregnant, mated but non-parturient, and GnRH-treated koalas was not significantly different overall, and, therefore, argues against the possibility of maternal recognition with respect to CL function and associated progesterone secretion. While the relevance of the peri-ovular progesterone pulse remains unclear, its elucidation may have a role in ovulation and early support of the CL and thus may have implications for successful AI.

In exploring koala pregnancy through the application of plasma proteomics, we report here the first proteome of koala plasma and reveal visual differences using a relaxed statistical test in plasma protein abundance at D2 and D34 in pregnant and non-pregnant koalas. However, we were unable to identify a suitable biomarker for pregnancy. Nevertheless, this preliminary study provides foundational information on the koala plasma proteome during pregnancy and the luteal phase, and identifies LRG1 and FN1 as candidate proteins for future targeted investigation into ovulation, fertilisation, embryonic development, and therefore pregnancy and/or embryonic loss. An ability to detect pregnancy in the koala has implications for reproductive management of captive breeding programmes [14] and in providing an important diagnostic for embryonic failure.

## Figures and Tables

**Table 1:** Mean (± SE) progesterone concentration (ng/mL) at each time point throughout gestation or luteal phase within the pregnant (*n* = 4 cycles from 3 females), mated but non-parturient (*n* = 4 cycles from 3 females), and GnRH-treated (*n* = 7) groups. Means in the same column are significantly different (*p* < 0.05) if not followed by any letter in common.

Time Points (D)	Pregnant	Mated But Non-Parturient	GnRH-Treated
0	20.55 ± 3.29 ^a^	16.06 ± 2.47 ^ad^	16.83 ± 2.10 ^a^
1	31.89 ± 6.86 ^ab^	36.13 ± 5.07 ^abd^	16.33 ± 2.70 ^a^
2	40.70 ± 3.33 ^ab^	42.07 ± 5.49 ^abd^	15.71 ± 1.83 ^a^
5	29.93 ± 2.48 ^ac^	28.97 ± 4.18 ^acd^	20.45 ± 1.61 ^ab^
10	26.53 ± 1.39 ^a^	29.22 ± 3.61 ^acd^	27.11 ± 2.06 ^ae^
15	30.83 ± 2.63 ^ac^	33.88 ± 3.89 ^ab^	39.09 ± 4.32 ^bcd^
20	43.14 ± 6.74 ^ab^	48.38 ± 5.31 ^bc^	52.63 ± 6.00 ^de^
25	59.71 ± 5.42 ^b^	60.76 ± 7.61 ^b^	66.81 ± 5.80 ^e^
30	56.28 ± 8.63 ^bc^	56.41 ± 9.11 ^bc^	53.64 ± 6.97 ^de^
35	35.53 ± 2.52 ^ab^	30.24 ± 8.72 ^ad^	33.14 ± 3.63 ^acd^
40	No data	17.28 ± 4.83 ^d^	18.80 ± 1.01 ^ac^
45	No data	16.85 ± 4.58 ^d^	17.87 ± 1.42 ^ac^

^a–e^ Means in the same column are significantly different (*p* < 0.05) if not followed by any letter in common.

**Table 2:** Top 15 most abundant proteins in female koala (*Phascolarctos cinereus*) plasma based on SWATH-MS.

Gene Symbol	Protein Name	Median Intensity	Known/Predicted Function
LOC110209798	Predicted serum albumin-like protein	23.19	Transport of various molecules; maintenance of colloid osmotic pressure in blood [23]
LOC11020404049	Predicted serum albumin-like protein	21.41	Transport of various molecules; maintenance of colloid osmotic pressure in blood [23]
HRG	Histidine-rich glycoprotein	21.14	Adaptor molecule for glycosaminoglycan-bearing surfaces; manages plasminogen activation [24]
APOA1	Apolipoprotein A1	18.99	Assists in reverse cholesterol transport to liver [25]
AHSG	Alpha-2-HS glycoprotein	18.58	Aids in biomineralisation of bone [26]
NDUFA12	NADH: ubiquinone oxidoreductase subunit A12	18.15	Assemble and stabilise extramembrane arm of the respiratory chain NADH dehydrogenase [27]
FGG	Fibrinogen gamma chain	17.69	Part of the fibrinogen dimeric protein that aids in blood coagulation, inflammation and wound healing, fibrinolysis, and cellular and matrix interactions [28]
FGB	Fibrinogen beta chain	17.39	Part of the fibrinogen dimeric protein that aids in blood coagulation, inflammation and wound healing, fibrinolysis, and cellular and matrix interactions [28]
FGA	Fibrinogen alpha chain	17.37	Part of the fibrinogen dimeric protein that aids in blood coagulation, inflammation and wound healing, fibrinolysis, and cellular and matrix interactions [28]
LOC110208182	Predicted lactotransferrin-like protein	17.25	Iron-binding; inhibits bacterial invasion [29]
LOC110197834	Predicted venom metalloproteinase inhibitor DM43-like protein	17.21	Inhibition of snake venom metalloproteinase [30]
PRPF40B	Pre-mRNA processing factor 40 homologue	17.36	Pre-mRNA splicing and transcription elongation [31]
A2M	Alpha-2-macroglobulin	16.83	Immune recognition and regulation; protease inhibitor [32]
LOC110220791	Predicted alpha-1-antiproteinase-like protein	16.10	Anti-inflammatory [33]
GC	GC vitamin D binding protein	15.88	Transport and storage of vitamin D; assists in inflammation and macrophage activation; scavenging of extracellular G-actin [34]

## Data Availability

The data underlying this article are available in the article and in its online Supplementary Materials. Proteomic data is available via ProteomeXchange under identifier PXD079795.

## Supplementary Materials

The following supporting information can be downloaded at: https://www.mdpi.com/article/10.3390/proteomes14030040/s1, Figure S1: Principal component analysis of proteomic data from day 2 post mating/injection in GnRH-treated (*n* = 3), mated but non-parturient (*n* = 3), and pregnant (*n* = 3) koala plasma; Figure S2: Principal component analysis of proteomic data from D34 post mating/injection in GnRH-treated (*n* = 3), mated but non-parturient (*n* = 3), and pregnant (*n* = 3) koala plasma; Table S1: Excel table of all proteins identified, protein abundance, and other essential proteomic information generated by DIA-NN; Table S2: Mean (± SE) LRG1 abundance in pregnant (*n* = 3), mated but non-parturient (*n* = 3), and GnRH-treated koalas (*n* = 3) at D0, D2, D9, D19, and D34; Table S3: Mean (± SE) FN1 abundance in pregnant (*n* = 3), mated but non-parturient (*n* = 3), and GnRH-treated koalas (*n* = 3) at D0, D2, D9, D19, and D34; Table S4: Proteins identified exclusively one treatment/time point but not identified in two or more biological replicates of in any biological replicate of another treatment/time point in comparisons of pregnant treatments (*n* = 3), mated but non-parturient treatments (*n* = 3), and GnRH induced ovulation treatments (*n* = 3) and at D0, D2, D9, D19, and D34 time points.
