# Supplementary material for: Comparative Analysis of Progesterone Secretion and Plasma Proteome Across the Pregnant and Non-Pregnant Luteal Phase of the Koala (Phascolarctos cinereus)
Source: Proteomes. 2026 Aug 6;14(3):40. doi: 10.3390/proteomes14030040 (PMC13511061; doi:10.3390/proteomes14030040)
Supplement: Supplementary file 1 [file proteomes-14-00040-s001.zip › proteomes-4393289-supplementary.pdf]

Supplementary material for Comparative analysis of progesterone secretion and plasma proteome across the pregnant and non-pregnant luteal phase of the koala (Phascolarctos cinereus)

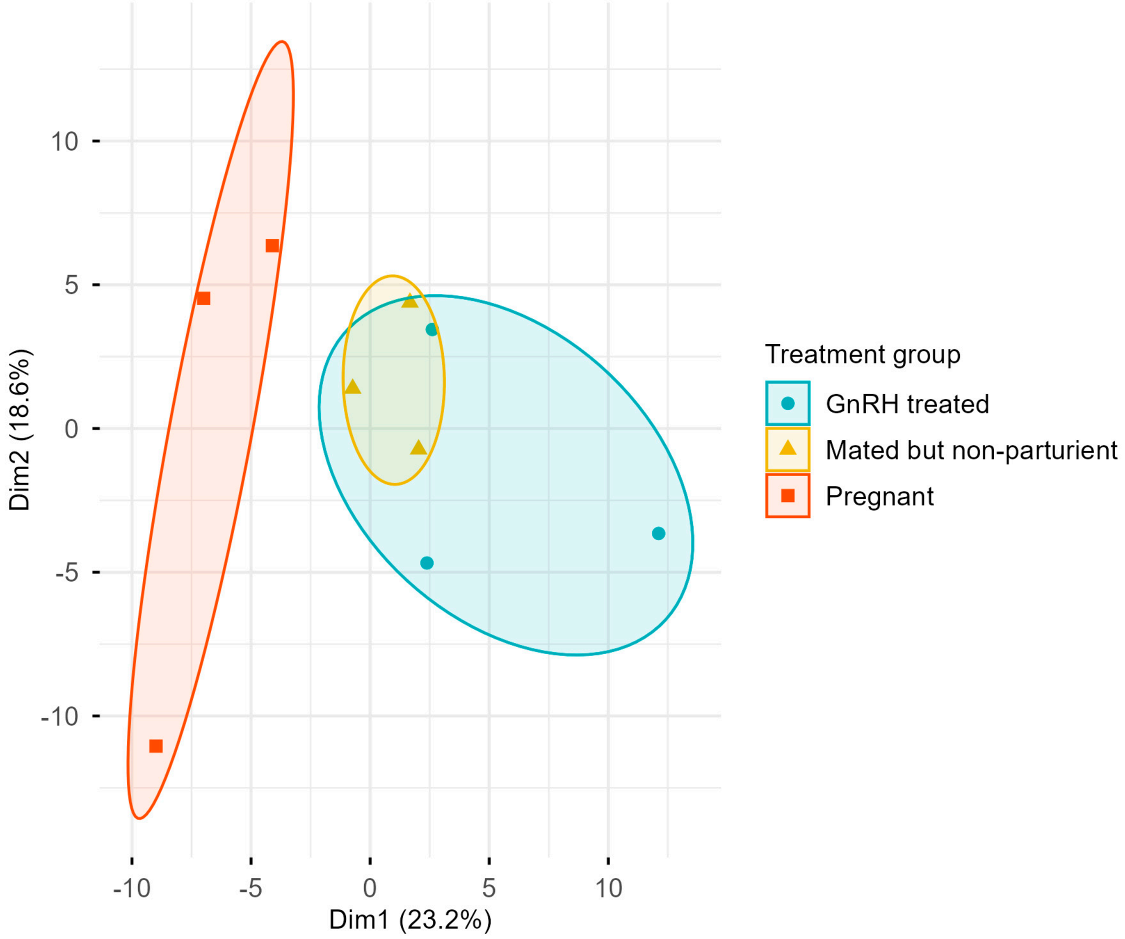

Figure S1. Principal component analysis of proteomic data from day 2 post mating/injection in GnRH treated (*n* = 3), mated but non-parturient (*n* = 3), and pregnant (*n* = 3) koala plasma.

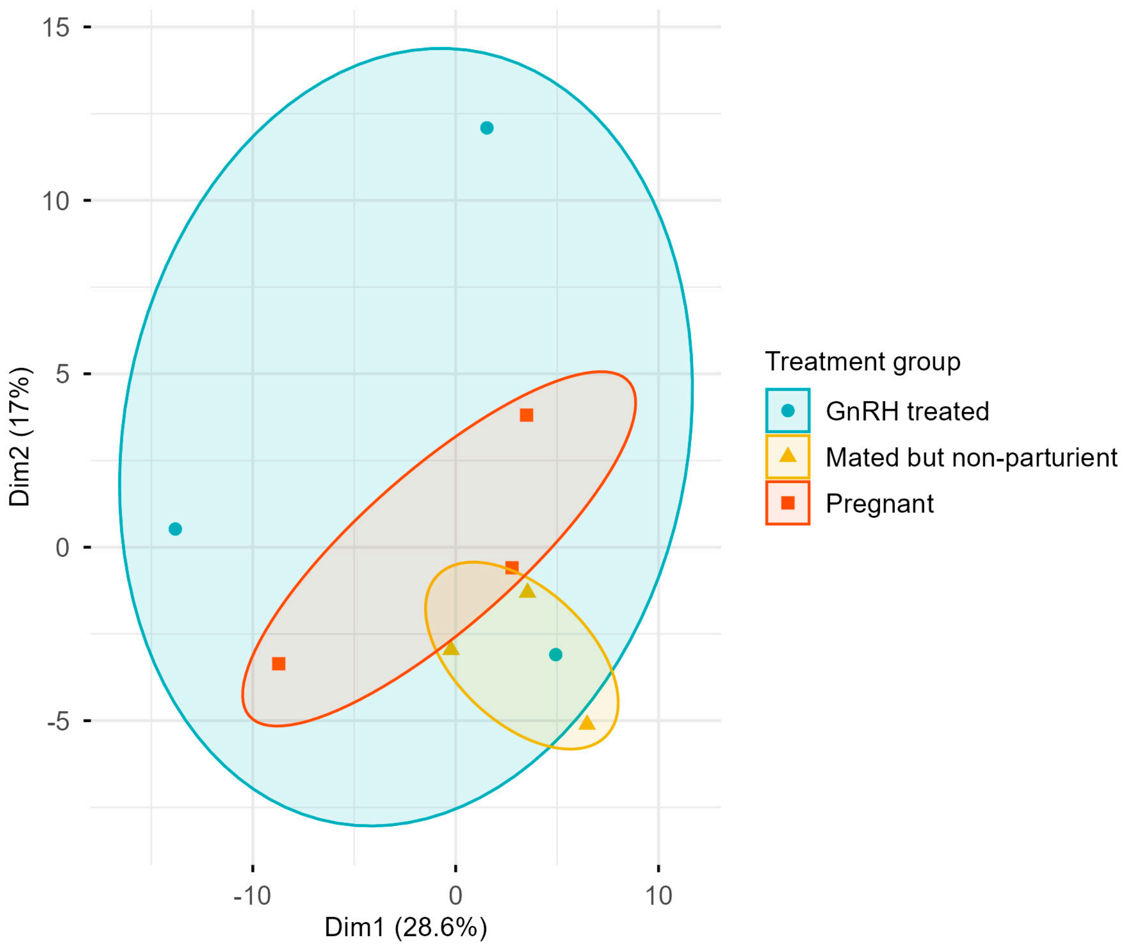

Figure S2. Principal component analysis of proteomic data from D34 post mating/injection in GnRH treated (*n* = 3), mated but non-parturient (*n* = 3), and pregnant (*n* = 3) koala plasma.

Table S2. Mean (± SE) LRG1 abundance in pregnant (*n* = 3), mated but non-parturient (*n* = 3), and GnRH treated koalas (*n* = 3) at D0, D2, D9, D19, and D34.

Timepoints (D)	Pregnant	Mated but non-parturient	GnRH treated
0	11.39 ± 0.07	11.58 ± 0.31	11.05 ± 0.03
2	12.66 ± 0.12[ab]	11.68 ± 0.15	11.11 ± 0.41[a]
9	11.14 ± 0.004[b]	11.34 ± 0.34	10.82 ± 0.13
19	11.07 ± 0.5[b]	11.45 ± 1.2	11.38 ± 0.06
34	12.04 ± 0.32	11.08 ± 0.01	10.85 ± 0.25

[a-b]Means are significantly different ($p < 0.05$) if followed by a letter in common.

Table S3. Mean (± SE) FN1 abundance in pregnant (*n* = 3), mated but non-parturient (*n* = 3), and GnRH treated koalas (*n* = 3) at D0, D2, D9, D19, and D34.

Timepoints (D)	Pregnant	Mated but non-parturient	GnRH treated
0	12.77 ± 0.69	13.05 ± 0.1	13.02 ± 0.2
2	13.24 ± 0.49	13.31 ± 0.32	13.5 ± 0.09
9	11.23 ± 1.34[a]	13.42 ± 0.77	13.11 ± 0.21
19	15.06 ± 1.94[a]	12.48 ± 0.18	13.11 ± 0.18
34	12.59 ± 0.41	12.74 ± 0.04	12.24 ± 0.43

[a]Means are significantly different ($p < 0.05$) if followed by a letter in common.

Table S4. Proteins identified exclusively one treatment/time point but not identified in two or more biological replicates of in any biological replicate of another treatment/time point in comparisons of pregnant treatments (*n* = 3), mated but non-parturient treatments (*n* = 3), and GnRH induced ovulation treatments (*n* = 3) and at D0, D2, D9, D19, and D34 timepoints.

Comparison		No. proteins – only first treatment/timepoint	No. proteins – only second treatment/timepoint	Total no. proteins only present in one treatment/timepoint or significantly different
**GnRH v Pregnant**	D0	1	7	8
	D2	6	1	8*
	D9	0	4	4
	D19	0	13	13
**GnRH v Mated but non-parturient**	D2	3	5	8
	D9	1	0	1
	D19	0	2	2
	D2	0	5	5
	D9	8	0	8
	D19	10	0	10
	D34	2	0	2
**D0 v D2**	GnRH	1	3	4
	Pregnant	13	0	13
	Mated but non-parturient	3	2	5
**D0 v D9**	GnRH	0	3	3
	Pregnant	0	4	4
	Mated but non-parturient	1	0	1
**D0 v D19**	GnRH	0	2	2
	Pregnant	2	5	7
	Mated but non-parturient	3	0	3
**D0 v D34**	GnRH	0	1	1
	Pregnant	2	4	6
	Mated but non-parturient	3	1	4
**D2 v D9**	Pregnant	0	19	20*
**D2 v D19**	Pregnant	0	19	20*
**D9 v D19**	Pregnant	0	3	4*

*Total number of proteins also includes significantly different (p <0.05) proteins that occur in both treatments/time point.
